# Mpox (Monkeypox) Virus and Its Co-Infection with HIV, Sexually Transmitted Infections, or Bacterial Superinfections: Double Whammy or a New Prime Culprit?

**DOI:** 10.3390/v16050784

**Published:** 2024-05-15

**Authors:** Benjamin M. Liu, Natella Y. Rakhmanina, Zhilong Yang, Michael I. Bukrinsky

**Affiliations:** 1 Division of Pathology and Laboratory Medicine, Children’s National Hospital, Washington, DC 20010, USA; 2 Department of Pediatrics, George Washington University School of Medicine and Health Sciences, Washington, DC 20010, USA; nrakhman@childrensnational.org; 3 Department of Pathology, George Washington University School of Medicine and Health Sciences, Washington, DC 20037, USA; 4 Department of Microbiology, Immunology & Tropical Medicine, George Washington University School of Medicine and Health Sciences, Washington, DC 20037, USA; mbukrins@gwu.edu; 5 Children’s National Research Institute, Washington, DC 20012, USA; 6 The District of Columbia Center for AIDS Research, Washington, DC 20052, USA; 7 Division of Infectious Diseases, Children’s National Hospital, Washington, DC 20010, USA; 8 Elizabeth Glaser Pediatric AIDS Foundation, Washington, DC 20005, USA; 9 Department of Veterinary Pathobiology, School of Veterinary Medicine & Biomedical Sciences, Texas A&M University, College Station, TX 77843, USA; zhilongyang@tamu.edu

**Keywords:** mpox, monkeypox virus, orthopoxvirus, co-infection, HIV, sexually transmitted infection, bacterial superinfection, trained immunity, diagnosis, treatment

## Abstract

Epidemiologic studies have established that mpox (formerly known as monkeypox) outbreaks worldwide in 2022–2023, due to Clade IIb mpox virus (MPXV), disproportionately affected gay, bisexual, and other men who have sex with men. More than 35% and 40% of the mpox cases suffer from co-infection with HIV and sexually transmitted infections (STIs) (e.g., *Chlamydia trachomatis, Neisseria gonorrhoeae, Treponema pallidum*, and herpes simplex virus), respectively. Bacterial superinfection can also occur. Co-infection of MPXV and other infectious agents may enhance disease severity, deteriorate outcomes, elongate the recovery process, and potentially contribute to the morbidity and mortality of the ensuing diseases. However, the interplays between MPXV and HIV, bacteria, other STI pathogens and host cells are poorly studied. There are many open questions regarding the impact of co-infections with HIV, STIs, or bacterial superinfections on the diagnosis and treatment of MPXV infections, including clinical and laboratory-confirmed mpox diagnosis, suboptimal treatment effectiveness, and induction of antiviral drug resistance. In this review article, we will discuss the progress and knowledge gaps in MPXV biology, antiviral therapy, pathogenesis of human MPXV and its co-infection with HIV, STIs, or bacterial superinfections, and the impact of the co-infections on the diagnosis and treatment of mpox disease. This review not only sheds light on the MPXV infection and co-infection of other etiologies but also calls for more research on MPXV life cycles and the molecular mechanisms of pathogenesis of co-infection of MPXV and other infectious agents, as well as research and development of a novel multiplex molecular testing panel for the detection of MPXV and other STI co-infections.

## 1. Introduction

Mpox (formerly known as monkeypox) virus (MPXV) belongs to the genus *Orthopoxvirus* (OPXV) and the family *Poxviridae*, which includes variola virus (causing smallpox), cowpox virus, vaccinia virus (VACV), and over 80 other species [1,2,3]. MPXV has long been recognized as a zoonosis that had been largely limited to regions of Africa, with few human-to-human transmissions prior to 2022 [1,4]. Since May 2022, rapidly spreading MPXV outbreaks due to human transmissions have been reported outside of Africa and evolved into a global health emergency, with more than 94,200 confirmed global cases in over 100 countries, including over 32,000 cases and 58 deaths in the United States [5,6,7,8,9,10].

Epidemiologic studies have established that more than 90% of the confirmed MPXV cases in the 2022–2023 outbreaks were gay, bisexual, and other men who have sex with men (MSM), who represent a high-risk population for both MPXV and HIV infection [5,6,7,8,9,10,11,12]. Co-infection with HIV and sexually transmitted infections (STIs) was observed among 38% and 41% of 1969 mpox cases in 8 U.S. jurisdictions, respectively, in a study by Curran et al. [7]. MPXV-infected cases are reported in children and are more likely to be severe [13]. We have recently diagnosed and managed three young adult mpox cases at the Children’s National Hospital in Washington DC, which led the nation in MPXV cases per capita, with >564 confirmed cases and 24 hospitalizations [14]. These three cases presented atypical manifestations, fewer rashes, and an absence of prodromal symptoms (unpublished data). Although there are no FDA-approved treatments specifically for MPXV infection, tecovirimat and brincidofovir approved for antiviral therapy of smallpox disease are recommended for antiviral treatment of mpox disease [15,16]. Tecovirimat was approved by the European Medicines Agency for the treatment of mpox disease in adults and children (body weight ≥ 13 kg) in January 2022 [15,17]. Although smallpox vaccines JYNNEOS and ACAM2000 can provide protection against MPXV infections, limited vaccine supply and the associated side effects and contraindications underscore the importance of developing novel preventive and therapeutic strategies to manage MPXV and/or HIV infections for high-risk populations [18,19].

In this review article, we will update the progress and knowledge gaps in MPXV biology, antiviral therapy, pathogenesis of human MPXV and its co-infection with HIV, STIs or bacterial superinfections, as well as the impact of the co-infections on the diagnosis and treatment of mpox disease. This review calls for research on outstanding questions in the MPXV life cycle and the molecular mechanisms of pathogenesis of co-infection of MPXV and other pathogens, MPXV drug resistance surveillance in MPXV- and HIV-co-infected cases for public health purposes, as well as research and development of a novel multiplex molecular testing panel for the detection of MPXV and the co-infections.

## 2. MPXV Biology

There are two clades (I and II) of MPXV that have been recognized, with Clade II further classified into two subclades: IIa and IIb. Clade I present in the Congo Basin was first recognized and is responsible for up to 10% of human mortality [1]. It is mainly transmitted from rodents to humans with limited human-to-human spread. Clade IIa was subsequently recognized in West Africa with an approximately 1% mortality, though it has a 95% nucleotide sequence identity to clade I [20]. Further, clade IIb, the current outbreak strain of MPXV, has sequence similarity to clade IIa and a much lower mortality in immunocompetent individuals [21]. Consistent with the severity of clinical disease in humans, Americo et al. demonstrated that there was a significant difference in MPXV virulence in CAST/EiJ mice in the order clade I > clade IIa > clade IIb [1].

**Figure 1:**
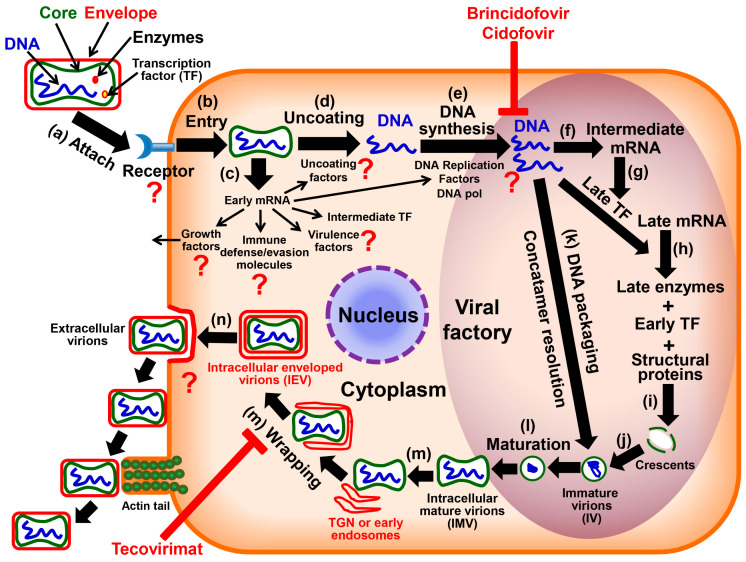
Life cycle of OPXVs including MPXV and molecular mechanisms of currently available antiviral strategies [2,15,17,22,23,24,38]. (**Upper**, **left**) MPXV is a DNA virus that has a membrane envelope surrounding an inner core containing viral DNA genome, transcription factors (TFs), and virion enzymes, e.g., DNA-dependent RNA polymerase and RNA-processing enzymes. MPXV replicates entirely in the cytoplasm. (**a**–**c**) After attachment and entry, viral early mRNAs are synthesized in the core and translated to generate non-structural proteins, including intermediate TFs and proteins necessary for immune defense, virulence (virulence factors), pathogenesis (pathogenic factors), and DNA replication. (**d**) The core wall opens and a nucleoprotein complex containing the genome is released from the core. (**e**) Replication of viral DNA genome is initiated, followed by the formation of viral factories. DNA synthesis is inhibited by cidofovir and brincidofovir. In the viral factory, the following steps take place, including (**f**–**h**) intermediate and late transcription, (**i**,**j**) crescent formation, (**k**) DNA packaging and concatamer resolution, as well as (**l**) virion maturation. Subsequently, (**m**) intracellular mature virions (IMV) become wrapped by a double membrane cisterna of the *trans*-Golgi network to form the intracellular enveloped virus (IEV), which is inhibited by tecovirimat. (**n**) IEV is capable of polymerizing actin tails, which facilitates the release (exocytosis) of the extracellular enveloped virus into the extracellular space. Of note, there are many outstanding questions that remain to be answered (question marks) in terms of molecular details during different steps of the MPXV life cycle.

Like other OPXVs, MPXV replicates entirely in the cytoplasm and has similar life cycle steps (Figure 1) [2,22,23,24,25,26], among which viral DNA replication and intracellular virion wrapping steps are targeted by brincidofovir and tecovirimat, respectively [15] (to be discussed in the next section). By employing electron microscopy, Paniz-Mondolfi et al. [27] recently observed typical enveloped virions with brick-shaped morphologies containing surface protrusions in MPXV and abundant melanosomes near viral assembly sites in skin lesions isolated from patients affected by the 2022–2023 outbreak. Of note, there are many outstanding questions that remain unanswered in the MPXV life cycle (Figure 1), for example, the cellular receptor of MPXV and the molecular details of virion morphogenesis and egress. Generally speaking, in response to viral invasion, the innate immune system presents a front line of host defenses armed with multi-layered mechanisms [2,28,29,30,31,32,33,34,35,36,37]. Of these, pattern recognition receptors, such as toll-like receptors (TLRs) and cyclic GMP-AMP synthase, sense OPXV nucleic acids, a major class of the viral pathogen-associated molecular pattern, triggering intracellular signaling pathways that activate the IFN production to restrict OPXV spread [2,36,37].

## 3. Antivirals against MPXV

Despite no MPXV-specific antivirals being approved, tecovirimat and brincidofovir (a prodrug of cidofovir), two FDA-approved, orally-bioavailable antivirals against smallpox in adults and children, are recommended for antiviral treatment of mpox disease, based on OPXV genome conservation, human safety profiles of these drugs, and their antiviral effectiveness in animal models [15,16,39]. Though lacking oral bioavailability, cidofovir is recommended by the Centers for Disease Control and Prevention (CDC) as one of the medical countermeasures available for the treatment of mpox due to its potent antiviral activities against OPXVs in in vitro and animal models and data on the effectiveness of brincidofovir [15,39]. As far as the mechanism of action is concerned, brincidofovir and cidofovir (a nucleoside analog) act as a DNA chain terminator to block DNA polymerase, thereby inhibiting OPXV DNA replication [15,39,40,41,42]. In contrast, tecovirimat suppresses the envelopment of intracellular mature virions and the formation of extracellular virus particles by inhibiting VP37 phospholipase (encoded by the *F13L* gene in VACV), thereby preventing the spread of the virus from an infected cell to other neighboring cells (Figure 1) [39,40,41,42].

Like other DNA viruses targeted by antivirals, e.g., hepatitis B virus, which accumulates antiviral drug resistance mutations [43,44], a potential issue that MPXV antiviral therapy may give rise to is the emergence of drug resistance, which has been a significant concern for the current antivirals [19,39]. Indeed, tecovirimat resistance has been identified in a small portion of patients with advanced HIV after treatment for periods of weeks to months [45]. Resistance to tecovirimat can develop in some patients who have subtherapeutic levels of the antiviral due to medication noncompliance or do not take the oral formulation within 30 min after eating full, fatty meals as indicated in the prescribing labeling [39]. Contrary to tecovirimat, MPXV seems less likely to develop resistance to brincidofovir or cidofovir partially due to the conservation of MPXV DNA polymerase [40,41]. However, mutations A314T and A684V in the VACV DNA polymerase (*E9L*) gene, which confer with cidofovir resistance, have been identified using in vitro selection model, which resides within the gene regions encoding the 3′-5′ exonuclease (A314T) and polymerase catalytic (A684V) domains [41].

**Figure 2:**
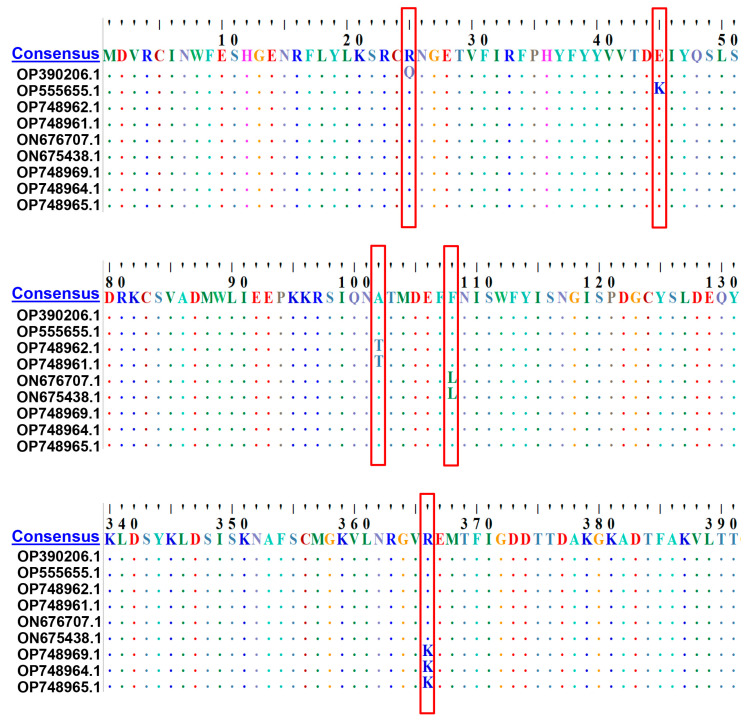
Identification of novel, potential brincidofovir/cidofovir antiviral resistance mutations in MPXV DNA polymerase (*F8L*) gene in the strains responsible for the 2022 mpox outbreak. A total of 230 MPXV *F8L* gene sequences were retrieved from whole genome sequences and aligned by ClustalW multiple alignment using BioEdit software, as reported elsewhere [46,47,48,49]. Among them, 110 and 120 sequences were isolated before and during the 2022 mpox outbreak, respectively. The consensus amino acid sequence of the *F8L* gene is shown at the top of the alignment. Although classic cidofovir resistance mutations [41] were not found among all MPXV sequences, 5 types of novel point mutations (R25Q (1/230), E45K (1/230), A102T (2/230), F108L (2/230), and R366K (3/230)) were identified among the 2022 mpox isolates (highlighted in red box).

The evolution of brincidofovir/cidofovir-resistant MPXV variants in patients is not fully understood. We aimed to examine the frequency and location of mutations in the MPXV DNA polymerase *F8L* gene (sharing ~98% homology with VACV *E9L*) and infer their impact on brincidofovir/cidofovir antiviral therapy. A total of 230 MPXV genome sequences were retrieved from the GenBank nucleotide sequence database, among which 110 and 120 strains were isolated before and during the 2022 mpox outbreak, respectively. Mutations in *F8L* gene sequences were identified and their potential impact on cidofovir efficacy was evaluated using structures of related viral and eukaryotic proteins and the structure prediction method AlphaFold. Phylogenetic analyses suggested that the included MPXV *F8L* gene sequences isolated before and during the 2022 outbreak did not cluster separately. Classic cidofovir resistance mutations A314T or A684V, as selected out in VACV [41], were not found among all MPXV sequences. However, 5 types of novel mutations were identified from 9 (7.5%, 9/120) sequences isolated during the 2022 mpox outbreak, including R25Q (1 strain), E45K (1 strain), A102T (2 strains), F108L (2 strains), and R366K (3 strains) (Figure 2). However, these mutations were not found in the included isolates before 2022. Given that topologically *F8L*-encoded DNA polymerase is responsible for ensuring DNA binding affinity of the replication complex, the newly identified mutations in the *F8L* gene may change the fidelity and processivity of MPXV DNA polymerase, thereby compromising its sensitivity to nucleoside inhibitors. These findings suggested that the circulating strains in the 2022 mpox outbreaks may accumulate novel, potential brincidofovir/cidofovir-resistant mutations in the MPXV DNA polymerase, whose clinical significance needs further investigation. Genomic surveillance will be warranted to monitor the evolution of resistant MPXV variants.

## 4. Pathogenesis of Human MPXV and Its Co-Infection with HIV, STIs, or Bacterial Superinfections

**Figure 3:**
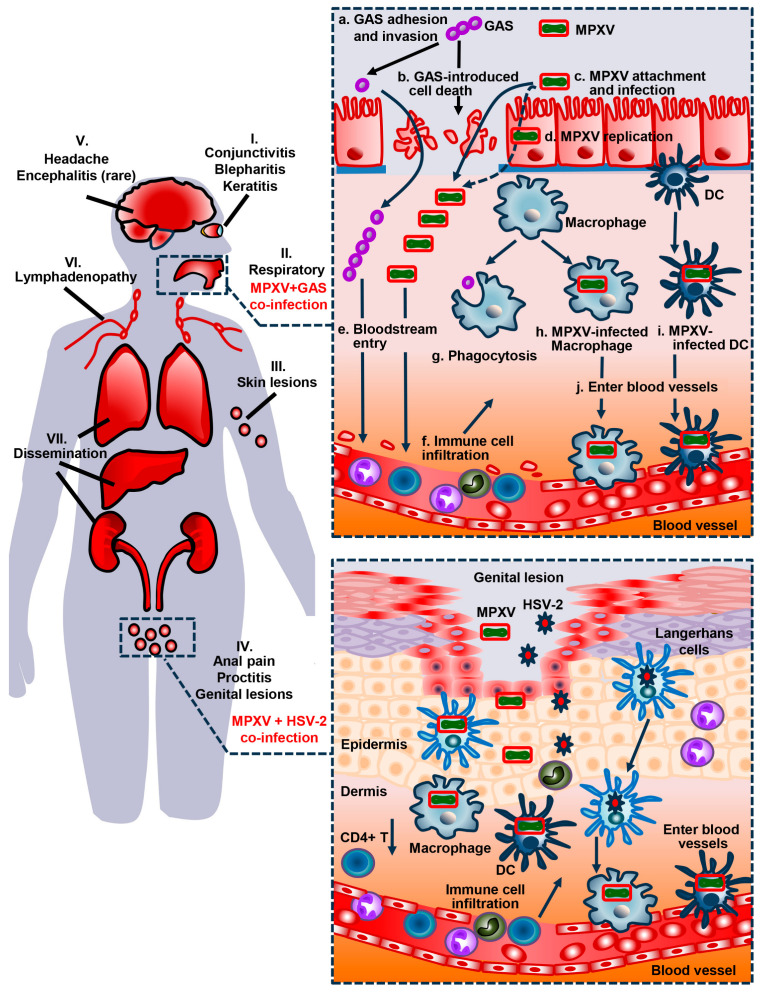
The pathogenesis of human MPXV and its co-infection with group A *Streptococcus* (GAS) and herpes simplex virus (HSV)-2. (**Left**) humans get primary MPXV infection via mucosa or skin, thereby leading to primary infection in (**I**) eye (e.g., conjunctivitis, blepharitis, and keratitis), (**II**) respiratory system, (**III**) skin, and (**IV**) genital sites (e.g., anal pain, proctitis, and genital lesions). (**V**) MPXV infection can cause neurologic symptoms, e.g., headache and, rarely, encephalitis. MPXV can disseminate via the lymph system and blood vessels, leading to (**VI**) lymphadenopathy and spreading to (**VII**) large organs, e.g., lungs, liver, and kidneys. (**Upper, right**) MPXV and GAS co-infection can be found in the upper respiratory tract. In this scenario, (**a**) GAS adheres and invades airway epithelial cells such as ciliated cells, and (**b**) induces cell death. (**c**) MPXV also attaches and infects ciliated cells, and (**d**) replicates in the primary infection sites. (**e**) GAS damages the blood vessel, facilitating entry of GAS and MPXV into the bloodstream. (**f**) Immune cells in peripheral blood start to infiltrate. (**g**) Macrophages initiate phagocytosis to engulf GAS. (**h**,**i**) Dendritic cells and macrophages can be infected with MPXV, and (**j**) enter blood vessels. (**Bottom, right**) MPXV and HSV-2 co-infection can occur in genital lesions, in which both MPXV and HSV-2 invade the genital skin and infect different types of cells, e.g., Langerhans, macrophages, and dendritic cells. The infection can lead to decreased CD4+ T cell count, infiltration of other immune cells, and viral dissemination via blood vessels.

Severe symptoms due to primary MPXV infection in humans implicate multiple organs and systems, which are aligned with the broad tropism of this virus (Figure 3, left) [2,50]. Humans get infected with MPXV via mucosa or skin, thereby leading to primary infection in the eye (e.g., conjunctivitis, blepharitis, and keratitis), respiratory system, skin, and genital sites (e.g., anal pain, proctitis, and genital lesions) [38,39,50,51,52,53]. MPXV infection can also cause neurologic symptoms, e.g., headache and, rarely, encephalitis. MPXV can disseminate via the lymphatic system and blood vessels, leading to lymphadenopathy and spreading to large organs, e.g., lungs, liver, and kidneys [39,50,51,52,53].

Co-infection of MPXV and HIV-1, STIs, or bacterial superinfections may enhance the severity, deteriorate outcomes of the symptoms, elongate the recovery process, and potentially contribute to morbidity and mortality of the ensuing disease [51,52,53]. First, a retrospective review of mpox (clade IIa) cases affected by a 2017–2018 outbreak in Nigeria suggested that HIV-1- and MPXV-coinfected patients suffered from more prolonged clinical course, extended lesions, and higher incidence of bacterial superinfections and genital ulcers, when compared with MPXV-positive but HIV-negative cases [54]. Second, MPXV and co-infection with STIs are reported in persons with HIV (PWH), e.g., *Chlamydia trachomatis*, *Neisseria gonorrhoeae*, *Treponema pallidum*, and herpes simplex virus (HSV) [7,53,55]. Among them, HSV-2 co-infection can exacerbate genital lesions, in which both MPXV and HSV-2 invade the skin and infect different types of cells, e.g., Langerhans, macrophages, and dendritic cells (Figure 3, bottom right). Furthermore, MPXV and bacterial superinfection, e.g., group A *Streptococcus* (GAS, i.e., *Streptococcus pyogenes*), can be found in the upper respiratory tract of both HIV-negative and HIV-positive cases [51] (Figure 3, upper right). GAS adheres and invades airway epithelial cells such as ciliated cells and induces cell death. MPXV also attaches and infects the same cell types and starts replication in the primary infection sites. GAS can damage the blood vessels, facilitating entry of GAS and MPXV into the bloodstream. Immune cells from peripheral blood infiltrate the infected tissue. Macrophages initiate phagocytosis to engulf GAS. Dendritic cells and macrophages can be infected with MPXV and enter blood vessels.

Notably, the co-infection of MPXV, STI pathogens, or bacteria superinfections in PWH could be attributed to behavioral factors, host immune status and responses, as well as virus–host interactions [1,5,6,7,8,9,10,11,12,56,57]. First, MSM-specific sexual exposures constitute a major risk factor for MPXV/HIV co-infection [5,6,7,8,9,10,11,12,56]. A recent systematic review [56] of 6345 cases affected by the 2022 mpox outbreaks (40.3% MPXV/HIV co-infection rate (2558/6345)) showed that 91.4% (5802/6345) of these cases were male patients, 56.2% (3259/5802) of whom had MSM sexual exposure, which may enhance the risk of infection of MPXV, HIV, STI pathogens, or bacteria superinfections. Second, weakened immune systems of PWH make them more vulnerable to infection with MPXV and other co-infections, e.g., STIs. Immunosuppressed/immunocompromised status, a low CD4+ T-cell count, and decreased immune response in PWH could give rise to the clinical severity of mpox disease, uncontrolled superinfection with other pathogens, and even mortality. For instance, two reported fatal cases with mpox and HIV co-infection had a CD4+ T-cells count < 200/μL [45,56]. In contrast, virus-specific T-cell responses can be detected among PWH participants after recovery from MPXV infection, indicating the involvement of T-cell responses in recovery from MPXV infection [57]. Third, generally speaking, innate immune responses, e.g., host restriction factors and cytokines/chemokines, and adaptive immune responses, e.g., T cell responses, play critical roles in sensing and restricting viral infections, including OPXVs [2,58,59,60]. However, MPXV has the ability to suppress cognate T cell activation and evade antiviral CD4+ and CD8+ T cell responses, thereby blocking inflammatory cytokine production and contributing to viral dissemination [61].

Before the 2022 MPXV outbreak, most of the PWH populations had not received the smallpox vaccine, which not only provides protection against MPXV but may also induce “trained immunity” to fend off other infections. The concept of trained immunity was introduced by Netea et al. [62,63,64] to describe the ability of innate immune cells (e.g., NK, dendritic cells, and monocytes/macrophages) to acquire an immunological memory to a “priming” pathogen (e.g., viruses, bacteria or fungi), a stimulus (e.g., TLR agonists, oxidized low-density lipoprotein, and aldosterone) or a vaccine (e.g., VACV-based smallpox vaccine), allowing them to trigger enhanced response to subsequent infection/stimulation by the same or unrelated agents/stimuli [65]. For example, innate immune responses associated with “trained immunity” persist after certain infections or vaccinations, e.g., with the smallpox vaccine. Weinstein et al. suggested that immunization with replicating smallpox vaccine, to some extent, might lead to a protective effect against subsequent HIV-1 infection [66], which could be explained by the concept of trained immunity. Compared with VACV-naive subjects, Weinstein et al. found that there was an up to five-fold reduction in the replication of CCR5-tropic, but not CXCR4-tropic, HIV-1 in the peripheral blood mononuclear cells (PBMCs) isolated from subjects vaccinated with replicating VACV smallpox vaccine Dryvax (Wyeth) 3–6 months before [66]. R5 HIV-1 replication in the PBMCs from VACV naive subjects, but not vaccinated subjects, was potentiated after adding autologous serum to the cell cultures, which is likely attributed to some activating effects in the serum of VACV naive subjects on the cultured PBMCs [66]. To establish trained immunity, metabolic pathways of innate immune cells are activated by the first stimulus, which lead to epigenetic changes and maintain the cell in a “trained” state. Innate immune memory or trained immunity can give rise either to stronger (enhancement) or weaker (suppression or tolerance) responses to subsequent infection or stimulation, which is dependent upon the strength and length of the first stimulation of the immune cells [65]. Similar to other functions of innate immunity, trained immunity has a broad range of responses and is quite different from the high specificity of classical memory T and B cells.

Similar to the concept of “trained immunity”, “heterologous immunity” is an immune response that is induced against a prior vaccination or exposure/infection with one pathogen but also can boost or weaken protective immunity against another unrelated pathogen, as suggested in studies with humans and mouse models [67,68,69]. Thanks to the cross-reactivity of T cell receptors, heterologous immunity to VACV infection has been experimentally shown in a mouse model that had been earlier exposed to infections with lymphocytic choriomeningitis virus, murine cytomegalovirus, Pichinde virus, or influenza A virus [67,68,69]. More research is needed to study the role of heterologous immunity and trained immunity in MPXV infection and co-infection with other etiologies.

## 5. The Clinical Impact of Co-Infections with HIV, STIs, or Bacterial Superinfections on the Diagnosis of MPXV Infections

With the advent of molecular microbiology, molecular testing has become the mainstay for infectious disease diagnostics with rapid turnaround time and high sensitivity, specificity, and accuracy [70,71,72]. Real-time PCR has played an important role in diagnosis of mpox infection in indicated populations in the 2022–2023 outbreaks. Per CDC guidelines [73], testing for MPXV infections is only recommended when there is a rash that is consistent with mpox. However, during the 2022–2023 mpox outbreaks, co-infections with different bacteria (e.g., GAS), STIs (e.g., syphilis), and other viruses (e.g., HSV-1, HSV-2, and varicella zoster virus) may complicate the differential diagnosis in adults as the co-infected organisms may present similar maculopapular rash, vesicles and pustule manifestations as MPXV does [51,52,53] (Figure 3), which may cause missing identification (false negativity) of MPXV if sampling the rashes or genital lesions due to co-infected pathogens (e.g., HSV-2) other than MPXV. For example, the symptoms of MPXV infection, e.g., lesions and rash, may overlap with other infections, such as GAS pharyngitis reported by Kaiser et al. [51]. Thus, the diagnosis of MPXV infection may be delayed. As another example in the pediatric world, enteroviruses (EV), coxsackievirus and human parechovirus with worldwide distribution may cause some symptoms that mimic the presentations of MPXV infection, e.g., hand-foot-and-mouth disease (HFMD) [71,74,75]. Similar to the findings by Fathi and Schmiedel [74], we tested skin swabs of atypical rashes from five pediatric cases with suspicion of mpox with Cepheid Xpert EV assay (off-label use) [72] between July and December 2022 at Children’s National Hospital in Washington, DC, which ended up being positive for EVs but negative for OPXV, suggesting atypical HFMD as an important differential diagnosis of mpox among pediatric populations.

Of note, a drawback of the currently available diagnostic real-time PCR assays employed in some reference CLIA labs (e.g., Mayo Clinic and ARUP Laboratories) in the U.S. during the 2022–2023 mpox outbreaks is that they are pan-OPXV real-time PCR, which fails to differentiate MPXV from other OPXVs, e.g., smallpox vaccine strains and molluscum contagiosum virus (MOCV) [76,77]. For example, co-infection with MOCV, an OPXV commonly responsible for self-limited infectious dermatosis among pediatric populations, may cause cross-reactions in the pan-OPXV PCR assays, when testing patients with suspicion of MPXV [77].

## 6. The Clinical Impact of Co-Infections with HIV, STIs, or Bacterial Superinfections on the Antiviral Treatment of MPXV Infections

Co-infections with different bacteria, STIs, and other viruses in mpox cases, especially those mpox and HIV co-infected cases, may complicate the management of the diseases, especially antiviral treatment [51,52,53]. First, co-infection with multiple organisms in mpox cases can be especially dangerous as the symptoms of the comorbidities can be exacerbated, making them more difficult to treat [56]. Second, co-infections with other organisms, e.g., STI pathogens, may affect the effectiveness of antiviral treatment for mpox and virus clearance. Opardija et al. recently reported delayed clearance of MPXV in a patient with co infection with secondary syphilis, despite a 14-day course of tecovirimat therapy [52]. However, in another cohort of patients with severe mpox who were treated with tecovirimat, treatment outcomes were not affected by HIV status [78]. Given the limited research in this area, it is important to acknowledge the caveat of limited data while highlighting potential outcomes on both fronts. Moreover, the delayed viral clearance due to co-infections of other pathogens may induce antiviral drug resistance and viral dissemination. Alarcón et al. reported a fatal mpox (clade IIb) and HIV co-infected cases (CD4+ T-cell count <35 per cubic millimeter) with recently treated syphilis, who developed tecovirimat resistance developed after 2 courses of oral tecovirimat [45]. Last but not least, antivirals against MPXV may have undesirable interactions with drugs used to treat co-infections [39]. For example, tecovirimat may lead to decreased levels of rilpivirine, a non-nucleoside reverse transcriptase inhibitor, whereas brincidofovir concentration may be elevated by protease inhibitors, cobicistat, and fostemsavir [39]. While cidofovir has minimal drug interactions, probenecid, which is co-administrated with cidofovir to minimize nephrotoxicity, has multiple drug interactions, e.g., zidovudine, beta-lactam antibiotics, diuretics, non-steroidal anti-inflammatory drugs, and angiotensin-converting enzyme inhibitors [39].

## 7. Conclusions

As an OPXV, clade IIb MPXV is responsible for the global 2022–2023 mpox outbreaks, with mild symptoms presented in most cases, though severe manifestations were observed in disseminated or immunocompromised cases. While there are no approved drugs specifically for MPXV, tecovirimat, brincidofovir, and cidofovir are recommended as medical countermeasures by the CDC.

More than 90% of the recently confirmed mpox cases were gay, bisexual, and other MSMs. Co-infection with HIV and STIs (e.g., *C. trachomatis*, *N. gonorrhoeae*, *T. pallidum,* and HSV) account for more than 35% and 40% of the mpox cases, respectively. Bacterial superinfection (e.g., GAS) can also occur. MSM-specific sexual behaviors constitute a major risk factor for MPXV/HIV co-infection. The co-infection of MPXV and other pathogens in PWH could be attributed to behavioral factors (e.g., MSM sexual exposure to MPXV, HIV, STI, and other pathogens), host immune status and responses (e.g., low CD4+ T cells, low trained or heterologous immunity), and virus-host interactions (e.g., virulent MPXV strains evading T cell responses).

Co-infections with HIV, STIs, or bacterial superinfections have a dramatic clinical impact on the pathogenesis, diagnosis, and treatment of MPXV infections. First, the co-infections of other pathogens may exacerbate symptoms and prolong illness. Second, co-infection of other pathogens mimicking mpox clinical presentations may lead to missed identification (e.g., false negative diagnostic MPXV PCR due to sampling HSV-2 genital lesions) or misidentification (e.g., false positive MPXV PCR due to cross-reactions with MOCV by pan-OPXV PCR) of MPXV, respectively. Third, the delayed viral clearance due to co-infections in mpox cases may induce antiviral drug resistance and viral dissemination.

## 8. Future Directions

This review calls for research on outstanding questions in the MPXV life cycle and the molecular mechanisms of pathogenesis of co-infection of MPXV and HIV, STIs, or bacterial superinfections. Research on the interplay between MPXV and other infectious agents and host cells in MPXV- and HIV-co-infected cases is warranted and will shed light on the mechanisms under which multiple organisms contribute to the pathogenesis in the context of mpox and HIV infections.

Drug resistance to tecovirimat, brincidofovir, and cidofovir should be monitored in MPXV- and HIV-co-infected cases for public health surveillance purposes. The current definition of drug resistance to these antivirals against MPXV is “clinical resistance” observed in the clinical response or MPXV viral load change in response to antiviral treatment. More sensitive and faster molecular testing for antiviral drug resistance mutations will be a valuable tool for screening and detection of “genotypic resistance” to predict “clinical resistance”. Moreover, identification and research of MPXV mutations conferring primary antiviral drug resistance (“primary drug-resistant” mutations) or compensated fitness (“secondary compensatory” mutations) are warranted.

MPXV-specific diagnostic real-time PCR assays, rather than pan-OPXV PCRs, should be used for definitive diagnosis of MPXV infection, which will allow for differentiation of MPXV from smallpox vaccine strains and MOCV. Considering the incidence of co-infection of MPXV and STIs, multiplex molecular testing panel for the detection of MPXV and co-infected STI pathogens (e.g., HSV-1, HSV-2, *T. pallidum*, *N. gonorrhoeae*, and *C. trachomatis*) in genital and anorectal lesions/ulcers is worthy development and clinical validation.

## Data Availability

Not applicable.
